# Deciphering Solution and Gas-Phase Interactions between Peptides and Lipids by Native Mass Spectrometry

**DOI:** 10.1021/acs.analchem.3c03428

**Published:** 2023-11-13

**Authors:** Til Kundlacz, Carla Schmidt

**Affiliations:** † Interdisciplinary Research Centre HALOmem, Institute of Biochemistry and Biotechnology, Charles Tanford Protein Centre, Martin Luther University Halle-Wittenberg, Kurt-Mothes-Str. 3a, 06120 Halle, Germany; ‡ Institute of Chemistry, Martin Luther University Halle-Wittenberg, von-Danckelmann-Platz 4, 06120 Halle, Germany; § Department of Chemistry—Biochemistry, Johannes Gutenberg University Mainz, Biocenter II, Hanns-Dieter-Hüsch-Weg 17, 55128 Mainz, Germany

## Abstract

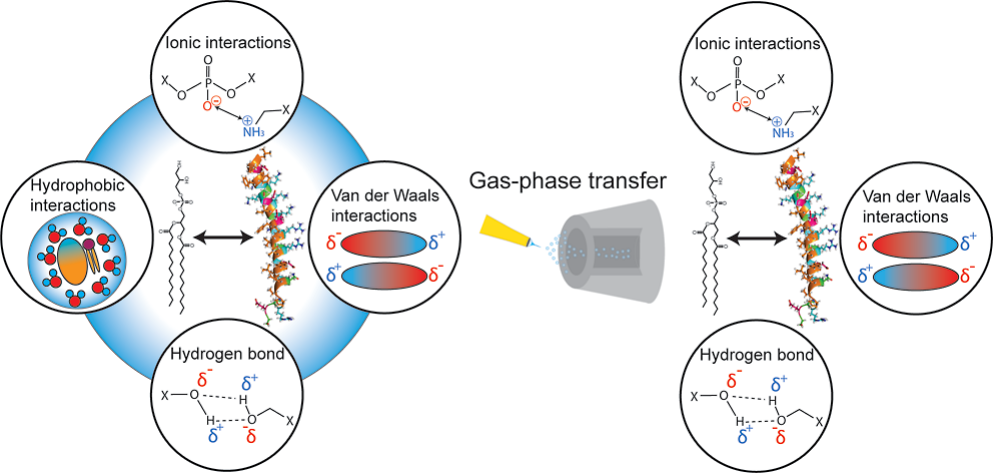

Many biological processes depend on the interactions between proteins and lipids. Accordingly, the analysis of protein–lipid complexes has become increasingly important. Native mass spectrometry is often used to identify and characterize specific protein–lipid interactions. However, it requires the transfer of the analytes into the gas phase, where electrostatic interactions are enhanced and hydrophobic interactions do not exist. Accordingly, the question remains whether interactions that are observed in the gas phase accurately reflect interactions that are formed in solution. Here, we systematically explore noncovalent interactions between the antimicrobial peptide LL-37 and glycerophospholipids containing different headgroups or varying in fatty acyl chain length. We observe differences in peak intensities for different peptide–lipid complexes, as well as their relative binding strength in the gas phase. Accordingly, we found that ion intensities and gas-phase stability correlate well for complexes formed by electrostatic interactions. Probing hydrophobic interactions by varying the length of fatty acyl chains, we detected differences in ion intensities based on hydrophobic interactions formed in solution. The relative binding strength of these peptide–lipid complexes revealed only minor differences originating from van der Waals interactions and different binding modes of lipid headgroups in solution. In summary, our results demonstrate that hydrophobic interactions are reflected by ion intensities, while electrostatic interactions, including van der Waals interactions, determine the gas-phase stability of complexes.

The interactions between membrane proteins and phospholipids rely on two noncovalent forces, namely electrostatic and hydrophobic forces. Whether electrostatic or hydrophobic forces dominate, strongly depends on the structure of the proteins and their lipid environment.^1,2^ Importantly, lipid membranes not only provide a stable environment for membrane proteins,^3−5^ they are also linked with their function and regulation.^4,6,7^ Investigating protein–lipid interactions, therefore, has gained importance over the past years.^8^

Of the available techniques, native mass spectrometry (MS) emerged as a well-suited tool to study protein–lipid interactions.^9−14^ Notably, native MS allows identification of the lipids that associate with the proteins as well as determination of their binding stoichiometry.^15−18^ Dissociation of the protein–lipid complexes through collision with an inert gas then provides insights into the binding strength of the lipids in the gas phase.^13^ However, native MS requires the transfer of biomolecules from solution into the gas phase, and the question of whether interactions observed in the gas phase reflect interactions formed in solution remains. Importantly, in the gas phase, interactions are dominated by electrostatic forces, while hydrophobic forces, which play a major role in solution, are under-represented.^19^ A systematic characterization of protein–lipid interactions in the gas phase, i.e., differentiating between electrostatic forces that apply in the gas phase and hydrophobic interactions that form in solution, is still missing.

Here, we explore the interactions of the antimicrobial peptide LL-37 with a range of glycerophospholipids containing different headgroups or varying infatty acyl chain lengths. Using native MS, we systematically probe noncovalent interactions in the gas phase and observe differences in the peak intensities of the complexes that form, providing us with a snapshot of the equilibrium in solution. Dissociation of these complexes through collisions with an inert gas further allows estimation of the relative binding strength of the lipids in the gas phase. In short, we found that interactions in solution are reflected in binding intensities, while the stability of peptide–lipid complexes in the gas phase can be assessed through collisional dissociation.

## Experimental Section

### Materials

Human LL-37 (trifluoroacetate salt, ≥95% purity) was purchased from Sigma-Aldrich (St. Louis, USA). The peptide was dissolved in phosphate-buffered saline (PBS) and stored at −20 °C. 1-*O*-(*n*-Octyl)-tetraethylene glycol (C8E4) was purchased from Glycon Biochem (Luckenwalde, Germany). 7.5 M ammonium acetate (AmAc) solution and PBS tablets were purchased from Sigma-Aldrich (St. Louis, USA). Chloroform (HPLC grade) was purchased from Alfa Aesar (Haverhill, USA). Methanol (LC/MS grade) was purchased from Fisher Scientific (Hampton, USA). 1,2-Dihexanoyl-*sn*-glycero-3-phospho-(1′-*rac*-glycerol) (PG 6:0/6:0), 1,2-dioctanoyl-*sn*-glycero-3-phospho-(1′-*rac*-glycerol) (PG 8:0/8:0), 1,2-didecanoyl-*sn*-glycero-3-phospho-(1′-*rac*-glycerol) (PG 10:0/10:0), 1,2-dilauroyl-*sn*-glycero-3-phospho-(1′-*rac*-glycerol) (12:0/12:0 PG), 1,2-dimyristoyl-*sn*-glycero-3-phospho-(1′-*rac*-glycerol) (PG 14:0/14:0), 1,2-dipalmitoyl-*sn*-glycero-3-phospho-(1′-*rac*-glycerol) (PG 16:0/16:0), 1,2-distearoyl-*sn*-glycero-3-phospho-(1′-*rac*-glycerol) (18:0/18:0 PG), 1,2-dimyristoyl-*sn*-glycero-3-phospho-l-serine (PS 14:0/14:0), 1,2-dimyristoyl-*sn*-glycero-3-phosphoethanolamine (PE 14:0/14:0), 1,2-dimyristoyl-*sn*-glycero-3-phosphocholine (PC 14:0/14:0), and 1,2-dimyristoyl-*sn*-glycero-3-phosphate (PA 14:0/14:0) were purchased from Avanti Polar Lipids (Alabaster, USA). All lipids were dissolved in 2:1 chloroform/methanol and stored in aliquots. For this, the solvent was evaporated under nitrogen, and dried lipids were overlaid with argon. Aliquots were stored at −20 °C. The lipid content was verified by phosphate analysis.^20^

### Preparation of Mixed Detergent–Lipid Micelles

For transfer of lipids during electrospray ionization or control experiments, mixed detergent–lipid micelles were prepared. For this, dried lipids were resuspended in 200 mM AmAc, pH 7.5 containing 0.5% (w/v) C8E4, followed by sonication for 30 min at 60 or 70 °C (PG 16:0/16:0) or 90 °C (PG 18:0/18:0).

### Dynamic Light Scattering

The mean hydrodynamic diameter of detergent–lipid micelles was determined using a Litesizer 500 particle size analyzer (Anton Paar, Graz, Austria). See the Supporting Information for details.

### Circular Dichroism Spectroscopy

Circular dichroism (CD) spectroscopy was performed using a J-810 spectropolarimeter (JASCO, Groß-Umstadt, Germany). See the Supporting Information for details.

### Sample Preparation for Native MS

LL-37 was transferred into 200 mM AmAc using Micro Bio-Spin P6-6 gel columns (Bio-Rad, Hercules, USA) according to the manufacturer’s instructions. Alternatively, LL-37 was transferred into AmAc using 3 kDa MWCO Amicon ultra centrifugal filters (Merck Millipore, Billerica, USA) according to the manufacturer’s instructions. The protein concentration was determined using the Bradford assay.^21^ Prior to native MS analysis, LL-37 was mixed with the detergent–lipid micelles to final concentrations of 20 μM LL-37, 25 μM lipid, and 0.5% (w/v) C8E4.

### Native MS

Native MS was performed on a Q-TOF Ultima mass spectrometer (Waters, Wilmslow, UK) modified for native MS.^22^ For each measurement, 3 μL of sample were loaded into a gold-coated borosilicate emitter needle produced in-house.^23^ Typical instrument settings were as follows: capillary voltage, 1.7 kV; capillary temperature, 80 °C; cone voltage, 35 V; collision voltage, 10–100 V; and RF lens voltage, 80 V. Dissociation of peptide–lipid complexes was achieved by increasing the collisional voltage from 10 to 100 V in 10 V increments. Number of replicates: 4 replicates for PG 6:0/6:0, 5 replicates for PG 8:0/8:0, PG 10:0/10:0 and PG 12:0/12:0, 6 replicates for PG 14:0/14:0, 8 replicates for PG 16:0/16:0, 9 replicates for 18:0/18:0 PG, 3 replicates for PS 14:0/14:0 and PA 14:0/14:0, and 4 replicates for PE 14:0/14:0 and PC 14:0/14:0.

### Data Analysis

The UniDec^24^ software was used for the deconvolution of unprocessed mass spectra using the following settings: *m*/*z* range, 820 to 3600; background subtraction, 20; bin size, 2.0; charge range, 1 to 8; mass range, 4400 to 6900 Da; peak full-width half-maximum, <1. The intensity (termed “height” in UniDec settings) of selected peaks was extracted after normalization of the mass spectra to the base peak. The extracted peak intensities of all charge states of LL-37–lipid complexes were summed up and divided by the extracted peak intensity of the total LL-37 monomer peaks, providing the relative abundance of LL-37–lipid complexes. Relative abundances obtained at different collisional voltages were divided by the relative abundance at 10 V. The data points were fitted using the Boltzmann sigmoidal function *y* = *A*_2_ + (1 – *A*_2_)/(1 + exp((*x* – *x*_0_)/d*x*)). CID50 values were obtained from this fit.

For visualization of mass spectra, the raw data was processed using MassLynx v4.1 (Waters, Wilmslow, UK). Accordingly, at least 70 scans were combined and smoothed twice with a smooth window of 20 using the Savitzky–Golay filter,^25^ followed by background subtraction applying a 30% reduction under the curve with a polynomial order of 3 and a tolerance of 0.01.

## Results and Discussion

### LL-37 as a Model Peptide

To systematically study peptide–lipid interactions in the gas phase, we used the antimicrobial peptide LL-37 as a model peptide. LL-37 is the only human antimicrobial peptide of the cathelicidin family;^26,27^ it defends the cell against bacteria or fungi by associating with their membranes causing destabilization and disruption of the membranes.^28,29^ LL-37 consists of a single amphipathic helix (Figure S1A) and, therefore, has a hydrophobic and a hydrophilic interface (Figure S1B). The hydrophobic interface allows interactions with the fatty acyl chains in the hydrophobic core of phospholipid bilayers, while the hydrophilic interface electrostatically interacts with the lipid headgroups.

Interactions between LL-37 and phospholipids depend on the correct folding of the amphipathic helix.^30^ We, therefore, first assessed the secondary structure of LL-37 by CD spectroscopy (Figure S1C). To reflect the conditions used in native MS, the secondary structure of LL-37 was analyzed in 20 mM AmAc in the presence and in the absence of 0.5% (w/v) C8E4. As high salt concentrations further effect secondary structure formation,^30^ LL-37 was also analyzed in PBS (Figure S1C). Note that lower AmAc concentrations were used during CD spectroscopy compared with native MS conditions to reduce the background absorption (20 mM instead of 200 mM AmAc). The CD spectrum of LL-37 in 20 mM AmAc shows a local minimum at 203 nm, indicating that the peptide is unstructured under these conditions.^31^ Accordingly, LL-37 was previously described to be disordered at low salt concentrations; a lack of anions showed the strongest effects in these experiments.^30^ Note that a higher AmAc concentration, as used in native MS experiments, might induce helix formation.

As C8E4 detergent was used for lipid transfer to LL-37, the peptide was also analyzed in the presence of 0.5% (w/v) C8E4. The corresponding CD spectrum shows local minima at 208 and 222 nm, which are characteristic for α-helical structures.^32^ Our findings indicate that 0.5% (w/v) C8E4 induces transitions of LL-37 from a random coil to an α-helix. This is in agreement with previous studies, showing that many antimicrobial peptides form α-helices in a hydrophobic environment.^33,34^ Our findings further suggest direct interactions between LL-37 and C8E4. Similarly, the CD spectrum of LL-37 in the presence of PBS showed local minima at 208 and 222 nm, confirming that α-helical structures form at higher salt concentrations.

### Characterization of Detergent–Lipid Micelles

Previous studies showed that detergent micelles stabilize integral membrane proteins in the gas phase^35−38^ and allow for lipid transfer from mixed detergent–lipid micelles.^13,39^ Here, we analyzed the interactions of the soluble peptide LL-37 with lipids by transferring lipids from detergent–lipid micelles as introduced recently.^40^ Although the underlying mechanism of the lipid transfer from detergent–lipid micelles is unknown, this procedure allows the study of lipid binding to soluble proteins and peptides. Importantly, individual lipids are transferred to the protein, providing us with the opportunity to explore the effects of individual lipid species.

To reach this goal, detergent–lipid micelles were prepared in 200 mM AmAc containing 0.5% (w/v) C8E4 and 25 μM of the respective phospholipids. To evaluate solubilization of the lipids, the particle size of these mixed micelles was determined by DLS and compared to C8E4 micelles (Figure S2). PC 14:0/14:0 was completely solubilized showing a particle size of approximately 5 nm similar to C8E4 micelles (Figure S2A). For PE 14:0/14:0, PA 14:0/14:0, and PS 14:0/14:0, additional distributions of large particle sizes were observed, suggesting that these lipids did not solubilize completely and formed higher aggregates. Note that the volume of these aggregates is <1% of the total volume, and these aggregates can, therefore, be neglected. Increasing the C8E4 concentration to 0.75% (w/v) and 2% (w/v) C8E4 resulted in complete solubilization of PS 14:0/14:0 as well as PE 14:0/14:0 and PA 14:0/14:0, respectively (Figure S2A). In contrast, PG lipid species ranging from PG 6:0/6:0 to PG 18:0/18:0 were completely solubilized at 0.5% (w/v) C8E4 with particle sizes similar to C8E4 micelles (Figure S2B); note that PG 16:0/16:0 and PG 18:0/18:0 have higher transition temperatures and required higher sonication temperatures for solubilization. Because populations of larger aggregates were neglectable (see above), the detergent concentration of 0.5% (w/v) was maintained for all measurements.

### Exploring Electrostatic Interactions of LL-37 with Different Lipid Headgroups

When interactions that form in solution are explored by the complexes that are observed in the gas phase, the experiments should be carefully designed, and the results should be interpreted with caution. While the equilibrium between the free and the ligand-bound peptide in solution can in principle be maintained in native MS experiments, there are several factors that influence the observed ratio between free and ligand-bound peptide ions:^41−44^ (i) optimized settings are required to reduce in-source dissociation (false negatives) and nonspecific complex formation due to high concentration of the ligands in the electrospray droplets (false positives). In addition, the position of the electrospray emitter might affect the ionization of intact complexes.^41^ We, therefore, fine-tuned instrument settings to maintain peptide–lipid complexes and kept them constant in all measurements. Note that oligomerization of LL-37 was described previously;^45^ we also observed oligomeric states of LL-37 in preliminary experiments; however, we fine-tuned instrumental parameters for the detection of peptide–lipid complexes, and LL-37 oligomers were absent or in low abundance in most mass spectra. (ii) Formation of complexes that is exclusively driven by hydrophobic interactions cannot be followed in the gas phase.^19,46^ Accordingly, we do not address interactions with purely hydrophobic molecules. (iii) The response factor describing the ionization and detection efficiency of the analytes depends on the size and structure of the analyte^43,47^ and should be comparable between ion species that are analyzed. Accordingly, the response factor of lipids depends on the chemical structure of the lipid headgroup and the length of the fatty acyl chains. In previous studies, the ionization efficiency was found to decrease with increasing length of the fatty acyl chains.^48,49^ However, these observations were made for lipids solubilized in chloroform/methanol, and ionization of LL-37–lipid complexes in native MS experiments differs from these experiments.^50^ As glycerophospholipids are relatively small molecules when compared to LL-37, the response factor of LL-37–lipid complexes is expected to be comparable to the response factor of free LL-37.

Taking these considerations into account, we first investigated noncovalent interactions of LL-37 with different glycerophospholipid headgroups by native MS. Specifically, we examined interactions of LL-37 with two zwitterionic lipids, namely, PC 14:0/14:0 and PE 14:0/14:0, as well as three negatively charged lipids, namely PA 14:0/14:0, PS 14:0/14:0, and PG 14:0/14:0. To this end, detergent–lipid micelles were prepared as described above and used to transfer the lipids onto LL-37. The formed LL-37–lipid complexes were then analyzed by native MS.

**Figure 1:**
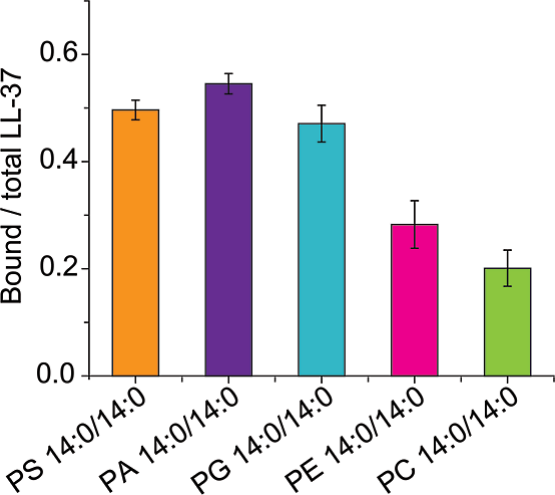
Interactions of LL-37 with lipids of different classes. Relative abundance of LL-37–lipid complexes containing PS 14:0/14:0 (orange), PA 14:0/14:0 (purple), PG 14:0/14:0 (light blue), PE 14:0/14:0 (pink), and PC 14:0/14:0 (light green).

The acquired mass spectra confirmed binding of all 5 phospholipids to LL-37 (Figure S3). Each mass spectrum revealed peaks corresponding in mass to LL-37 with up to 3 associated lipids. Importantly, peak intensities for complexes with zwitterionic lipids, e.g., PC 14:0/14:0, were comparably lower than those observed for complexes with negatively charged lipids, e.g., PS 14:0/14:0 (Figure S3). To determine binding preferences of the different lipid classes, relative abundances of detected complexes were determined as described (Experimental Section) and compared (Figure 1). Accordingly, the relative abundance of LL-37–lipid complexes that contain negatively charged lipids (PG 14:0/14:0, PA 14:0/14:0 or PS 14:0/14:0) was approximately 50%, while the relative abundance of complexes that include zwitterionic lipids (PC 14:0/14:0 or PE 14:0/14:0) was <30%. The lipid binding preference for the different negatively charged lipids was comparable, and only a slight increase of PG 14:0/14:0 < PS 14:0/14:0 < PA 14:0/14:0 was observed. The zwitterionic lipid PE 14:0/14:0 showed slightly higher binding than PC 14:0/14:0. The formation of more hydrogen bonds between the amine group of PE 14:0/14:0 and LL-37 might stabilize the complexes in the gas phase; apart from the phosphate group, the predominantly ionic PC headgroup is not involved in hydrogen bonding. Our results are in agreement with previous findings that revealed a selectivity of LL-37 for negatively charged phospholipids.^51−54^ However, it is important to take into account that the approach followed here is limited to the transfer of lipids from detergent–lipid micelles, and the effects of chain packing or lipid–lipid interactions as present in lipid bilayers cannot be assessed.

### Electrostatic Interactions Determine the Gas-Phase Stability

**Figure 2:**
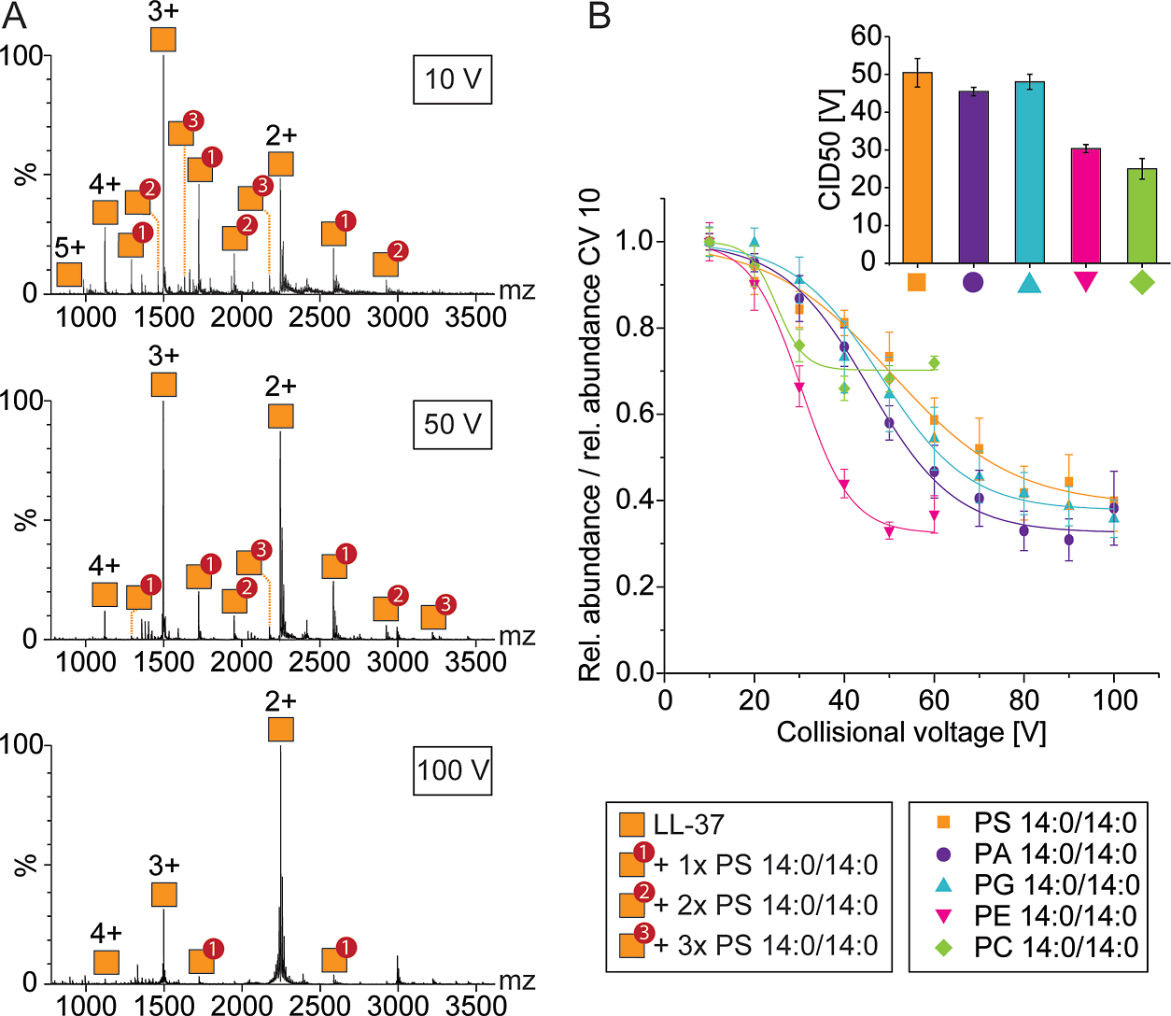
Probing electrostatic interactions in the gas phase by collisional dissociation. (A) Native MS of LL-37 in the presence of C8E4 with PS 14:0/14:0 at different collisional voltages. Charge states and lipid adducts are assigned. Masses of LL-37–lipid complexes are given in Table S1. (B) Collision-induced dissociation of LL-37–lipid complexes. The relative abundance of LL-37–lipid complexes determined for each collisional voltage (CV) was divided by the relative abundance observed at 10 V. The data points were fitted to sigmoidal functions. The CID50 values are plotted for each lipid class.

Having investigated the formation of LL-37–lipid complexes in solution, we explored the stability of the complexes in the gas phase by collisional dissociation. For this, the collisional voltage in the collision cell was increased from 10 to 100 V in 10 V increments. The peak intensities of the LL-37–lipid complexes decreased with increasing collision voltages (Figure 2A). Comparing the decrease in intensity of different LL-37–lipid complexes (Figure 2B), we estimate the relative binding strength of the lipids in the gas phase. Note that the strength of interactions in the gas phase differs from the strength in solution.^55^ For instance, the strength of Coulombic interactions increases by a factor of 80 and van der Waals interactions by a factor of 6400^56^ when transferring molecules from solution into the gas phase. Furthermore, desolvation during electrospray ionization not only leads to the loss of hydrophobic interactions but also strengthens hydrogen bonding by eliminating competition with surrounding water molecules.^57^ Consequently, rearrangements of the complexes to a gas-phase conformation are possible.^58,59^

Given that the lipids investigated here contain identical fatty acyl chains, differences in binding can be deduced from electrostatic interactions of the lipid headgroups. Since electrostatic interactions are equally enhanced in the gas phase, we are able to compare the relative binding strength in the gas phase and in solution. For this, peak intensities of LL-37–lipid complexes at different collisional voltages were extracted, and the relative abundances of the LL-37–lipid complexes were calculated (see the Experimental Section). The relative abundance obtained for each collisional voltage was then divided by the relative abundance at 10 V. LL-37–lipid complexes containing zwitterionic lipids, i.e., PC 14:0/14:0 and PE 14:0/14:0 showed the lowest binding strength with the lowest intensity at approximately 40 and 50 V, respectively (Figures 2B and S4). Note that the background signal in these measurements was comparably high (Figure S4). The negatively charged lipids, on the contrary, showed high binding strengths with similar dissociation behavior. Accordingly, LL-37–lipid complexes containing PS 14:0/14:0 and PA 14:0/14:0 showed lowest intensities at collisional voltages of approximately 80 V and those containing PG 14:0/14:0 at approximately 100 V. These findings are supported by the CID50 values, i.e., the collisional voltage at which 50% of the complex’ intensity is reached. Accordingly, gas-phase stability is reduced for zwitterionic lipids compared with their negatively charged counterparts. These results are in agreement with our own (see above) and previous findings that showed preferred interactions of LL-37 with negatively charged lipids.^51−54^

### Exploring Hydrophobic Interactions between LL-37 and Fatty Acyl Chains

We next explored hydrophobic interactions of LL-37 with lipids that differ in fatty acyl chain length. For this, we used a range of PG lipids with fatty acyl chains that increased in length by two methylene groups per fatty acyl chain and increment, namely, PG 6:0/6:0, PG 8:0/8:0, PG 10:0/10:0, PG 12:0/12:0, PG 14:0/14:0, PG 16:0/16:0, and PG 18:0/18:0. Native mass spectra confirmed binding of all PG lipids to LL-37 under the conditions applied here (Figure S5). Again, the mass spectra revealed LL-37–lipid complexes with up to 3 associated lipids (Figure S5).

**Figure 3:**
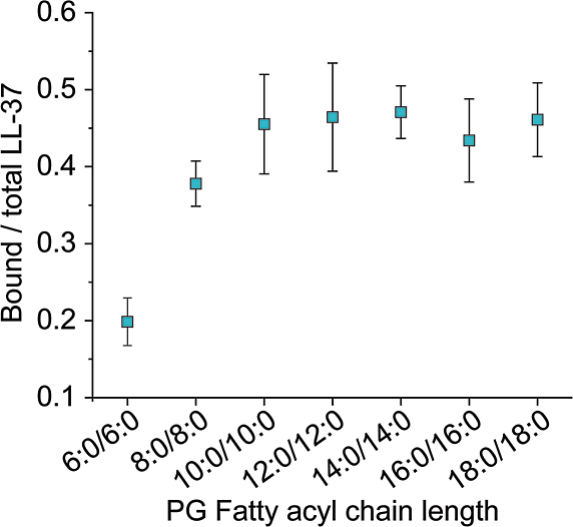
Formation of LL-37–lipid complexes including PG lipids with different fatty acyl chain lengths. The normalized sum of intensities of LL-37–lipid complexes with PG lipids varying in fatty acyl chain length is shown.

To determine lipid binding preferences, we next evaluated the relative abundance of complexes that were assembled from each LL-37–PG combination (Figure 3). Native mass spectra revealed a lower relative abundance of complexes containing lipids with short fatty acyl chains. For instance, complexes that contained PG 6:0/6:0 showed an intensity of approximately 20% when compared to total LL-37. In contrast, complexes that contained lipids with longer fatty acyl chains showed higher relative abundances of approximately 45% when compared with total LL-37 (Figure 3). Importantly, only smaller differences were observed between PG 10:0/10:0 and PG 18:0/18:0 lipids; the relative abundance for lipids with fatty acyl chain length >10 carbon atoms reached a plateau.

The increase in the relative abundance of complexes that assemble from PG 6:0/6:0 to PG 10:0/10:0 is likely due to an increase in hydrophobic interactions in solution. The fact that the relative abundance of complexes containing PG lipids with fatty acyl chains >10 carbon atoms does not increase above a certain limit was surprising and might have different reasons: first, the solubility of these lipids in aqueous solutions is low due to their hydrophobicity. Note that DLS analysis revealed complete solubility of these lipids as indicated by micelle formation (Figure S2); however, potential lipid aggregates might not be captured in these measurements due to precipitation. Another explanation might be that LL-37 prefers interactions with fatty acyl chains of a defined length. Due to its amphiphilic structure, the maximum number of hydrophobic contacts likely depends on the size of the hydrophobic interface of LL-37 and longer fatty acyl chains do not increase hydrophobic contacts. Again, the absence of a phospholipid membrane might affect these interactions (see above). As the mechanism behind the transfer of lipids from detergent–lipid micelles remains elusive, the hydrophobicity of the lipids might also influence their transfer. Nonetheless, the increase in relative abundance when forming complexes that contain lipids with longer fatty acyl chains suggests that more hydrophobic interactions are formed in solution resulting in increased complex formation; even though only electrostatic interactions are stabilized in the gas phase, the higher relative abundance of the complexes in solution is visualized in native MS experiments.

### Probing Hydrophobic and van der Waals Interactions in the Gas Phase

**Figure 4:**
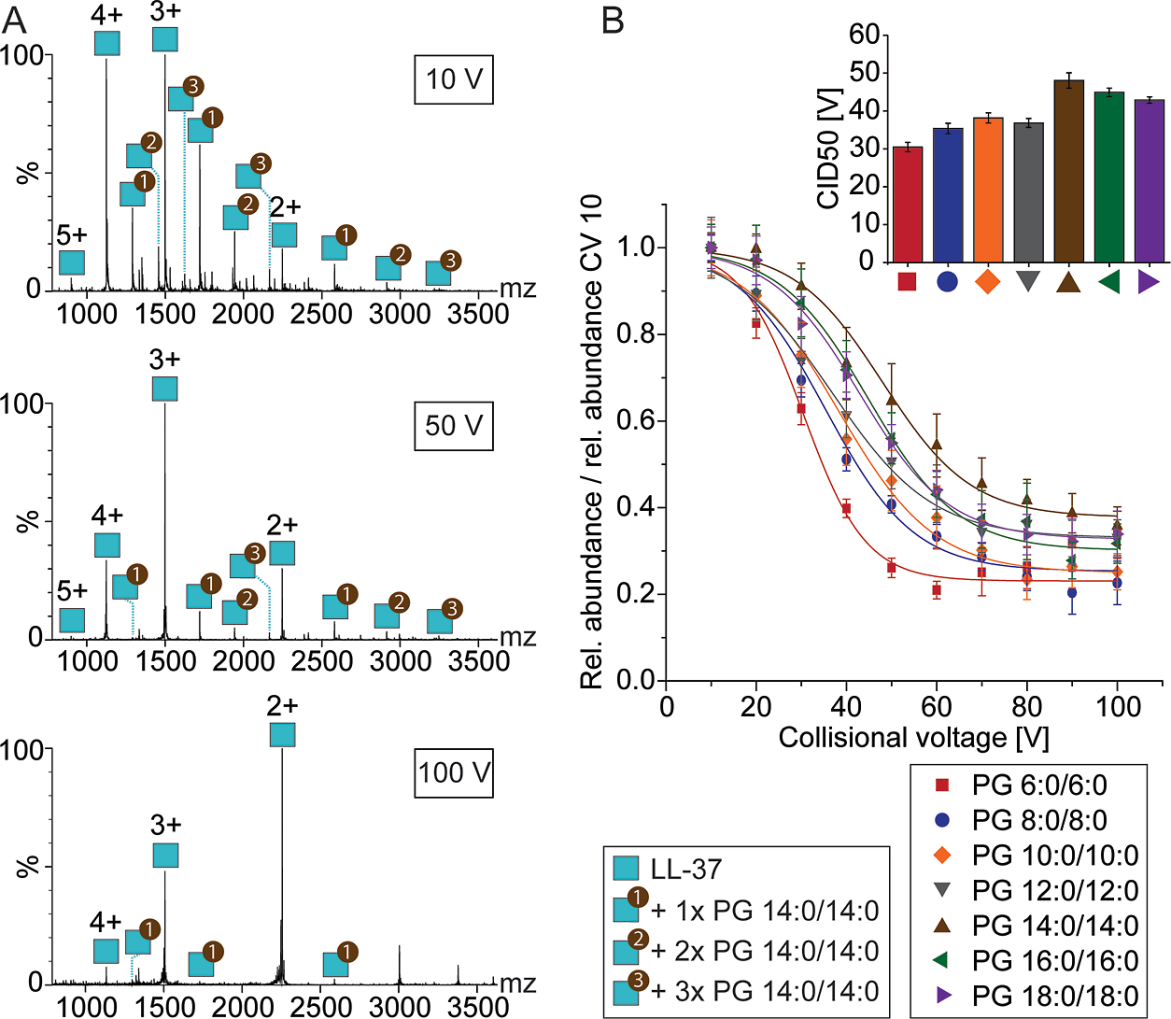
Probing hydrophobic and van der Waals interactions by gas-phase dissociation. (A) Native MS of LL-37 in the presence of C8E4 with PG 14:0/14:0 at different collisional voltages. Charge states and lipid adducts are assigned. Masses of LL-37–lipid complexes are given in Table S1. (B) Collision-induced dissociation of LL-37–lipid complexes. The relative abundance of LL-37–lipid complexes obtained at each collisional voltage (CV) was divided by the relative abundance determined at 10 V. The data points were fitted to sigmoidal functions. The CID50 values are plotted for each lipid class.

To gain detailed insights into the interactions between LL-37 and PG lipids that differ in fatty acyl chain length, the formed complexes were dissociated in the gas phase as described above. By correlating the observed binding strength in the gas phase with the detected ion intensities, we are able to distinguish between interactions that drive complex formation in solution and interactions that stabilize the peptide–lipid complexes in the gas phase. For this, the peak intensities of LL-37–lipid complexes at different collisional voltages were extracted to determine the binding strength in the gas phase. Again, peak intensities of LL-37–lipid complexes decrease with increasing collisional voltage; Figure 4A shows an example. The relative abundances of LL-37–lipid complexes at different collisional voltages were subsequently compared (Figure 4B). We observed a similar binding strength for most PG lipids dissociating at approximately 80 to 100 V. Note that PG 6:0/6:0, the lipid with the shortest fatty acyl chain, dissociated at a lower collisional voltage of approximately 60 V. The binding strength of PG 6:0/6:0 might be underestimated due to the low intensity of the complex at low collisional voltages (Figure S5) resulting in a lower signal-to-noise ratio at higher collisional voltages. Only minor differences in binding strength were observed between PG lipids with longer fatty acyl chains. Importantly, a slight increase in the binding strength was observed for increasing fatty acyl chain length (i.e., PG 8:0/8:0 < PG 10:0/10:0 < PG 12:0/12:0 < PG 16:0/16:0 ≈ PG 18:0/18:0). Surprisingly, PG 14:0/14:0 showed the highest binding strength dissociating at approximately 100 V. We assume that the higher binding strength for complexes containing lipids with longer fatty acyl chains results from van der Waals interactions. Accordingly, we assume that the fatty acyl chains of PG lipids are in close contact with LL-37 in the gas phase. We conclude that the binding strength of the same lipid class in the gas phase mostly relies on interactions with the headgroups. Slight differences might be explained by van der Waals interactions that are higher for longer fatty acyl chains than for short-chain lipids. Nonetheless, differences in the binding modes of PG lipids with long or short fatty acyl chains in solution should also be considered; binding of the lipids in different local environments on the protein surface might cause differences in the electrostatic interactions formed through the lipid headgroup and consequently their stabilization in the gas phase.

## Conclusions

In this study, we systematically investigated the interactions of the antimicrobial peptide LL-37 with glycerophospholipids containing different headgroups and varying fatty acyl chain lengths. For this, lipids were transferred from C8E4–lipid micelles as previously described.^40^ Using native MS, we explored whether interactions that form in solution are reflected in the ion intensities and complex stability in the gas phase.

We found that interactions between LL-37 and negatively charged lipids are preferred; this observation is reflected in the ion intensities and complex stability. Accordingly, electrostatic interactions with the lipid headgroups are responsible for the binding specificity in solution and, furthermore, determine the relative binding strength in the gas phase. Note that the binding strength in solution is less affected by experimental factors such as the response factor and might, therefore, be a more reliable measure for determining the specificity when electrostatic interactions are predominant. Native MS, therefore, is well-suited to investigate electrostatic interactions formed in solution and stabilized in the gas phase.

Probing interactions between LL-37 and PG lipids varying in fatty acyl chain length, we further explored hydrophobic interactions. Even though hydrophobic interactions are not stabilized in the gas phase, we observed differences in the peak intensity of complexes between LL-37 and PG lipids containing shorter or longer fatty acyl chains. We relate these differences to differences in the hydrophobic interactions in solution. Differences in the dissociation of the complexes in the gas phase are further attributed to van der Waals interactions, which, in addition to other electrostatic interactions, contribute to complex stability in the gas phase. The increase in van der Waals interactions suggests the presence of direct contacts between the fatty acyl chains and LL-37 in the gas phase, an observation that cannot be verified by MS, however, might change our understanding of protein structures and protein complexes in the gas phase. Identifying the mechanism of lipid transfer from detergent–lipid micelles to proteins in future studies will further increase our knowledge of protein–lipid and protein–detergent interactions in solution and in the gas phase.

In summary, we show that the peak intensity observed in native mass spectra does not necessarily correlate with the stability of the assemblies in the gas phase. Nonetheless, native MS is a well-suited tool to evaluate interactions that are formed in solution and observed in the gas phase.

## Supporting Information Available

Experimental details on dynamic light scattering and CD spectroscopy; structure of LL-37, DLS analysis of detergent–lipid micelles, exploring electrostatic interactions with lipid headgroups, dissociation of LL37–PE and LL-37–PC complexes, exploring hydrophobic interactions of LL-37 with fatty acyl chains; masses of LL-37–lipid complexes (PDF)
