# Supplementary material for: Deciphering Solution and Gas-Phase Interactions between Peptides and Lipids by Native Mass Spectrometry
Source: Anal Chem. 2023 Nov 13;95(47):17292–9. doi: 10.1021/acs.analchem.3c03428 (PMC10688224; doi:10.1021/acs.analchem.3c03428)
Supplement: Supplementary file 1 — ac3c03428_si_001.pdf [file ac3c03428_si_001.pdf]

Supporting Information

Deciphering Solution and Gas-Phase Interactions between Peptides and Lipids by Native Mass Spectrometry

Til Kundlacz[1,2], Carla Schmidt[1,3*]

[1]Interdisciplinary Research Centre HALOmem, Institute of Biochemistry and Biotechnology, Charles Tanford Protein Centre, Martin Luther University Halle-Wittenberg, Kurt-Mothes-Str. 3a, 06120 Halle, Germany

[2]Institute of Chemistry, Martin Luther University Halle-Wittenberg, Von-Danckelmann-Platz 4, 06120 Halle, Germany

[3]Johannes Gutenberg University Mainz, Department of Chemistry – Biochemistry, Biocenter II, Hanns-Dieter-Hüsch-Weg 17, 55128 Mainz, Germany

*Correspondence: carla.schmidt@uni-mainz.de

Table of Contents Page

1. Supporting Methods S3

1.1. Dynamic light scattering S3

1.2. Circular dichroism spectroscopy S3

2. Supporting Figures S4

Figure S1: Structure of LL-37. .... S4

Figure S2: DLS analysis of detergent-lipid micelles. S5

Figure S3: Exploring electrostatic interactions with lipid headgroups. S6

Figure S4: Dissociation of LL-37-PE and LL-37-PC complexes. S7

Figure S5: Exploring hydrophobic interactions of LL-37 with fatty acyl chains. S8

3. Supporting Tables S9

Table S1: Masses of LL-37 and LL-37-lipid complexes determined by native MS. S9

4. Supporting References S11

1. Supporting Methods

1.1. Dynamic light scattering

The mean hydrodynamic diameter of detergent-lipid micelles was determined using a Litesizer 500 particle size analyzer (Anton Paar, Graz, Austria). For this, 100 µl of detergent-lipid micelles were prepared as described and analyzed in a 3 x 3 mm ultra-micro cuvette (Hellma Analytics, Müllheim, Germany). The particles were irradiated with a semiconductor laser diode at 658 nm. The following instrument settings were applied: measuring angle, side scatter (90°); temperature, 25 °C; measurement time, automatic; filter, automatic; focus, automatic; material, phospholipids; solvent, 154 mM NaCl. The mean hydrodynamic diameter was determined from size distribution histograms using the software Kalliope (Anton Paar, Graz, Austria).

1.2. Circular dichroism spectroscopy

Circular dichroism (CD) spectroscopy was performed using a J-810 spectropolarimeter (JASCO, Groß-Umstadt, Germany). For this, 50 µl of a 1 mg/ml solution of LL-37 in 1 x PBS as well as in 20 mM AmAc in the presence and absence of 0.5 % (w/v) C8E4 were analyzed in a 0.1 mm quartz cuvette at 20 °C. The following instrument parameters were applied: wavelength, 190-240 nm; scanning mode, continued; scan number, 64 scans; scan speed, 50 nm/min; response, 1 s; and data pitch, 1 nm. The raw data was reduced to data points at HT voltage below 600 V as the signal to noise ratio is lower at high dynode voltages. CD spectra were smoothed using a binomial filter and the reference spectrum of the buffer was subtracted using the Spectra Manager software (JASCO). The ellipticity was converted to mean residue ellipticity (Δε) as described previously[1].

2. Supporting Figures

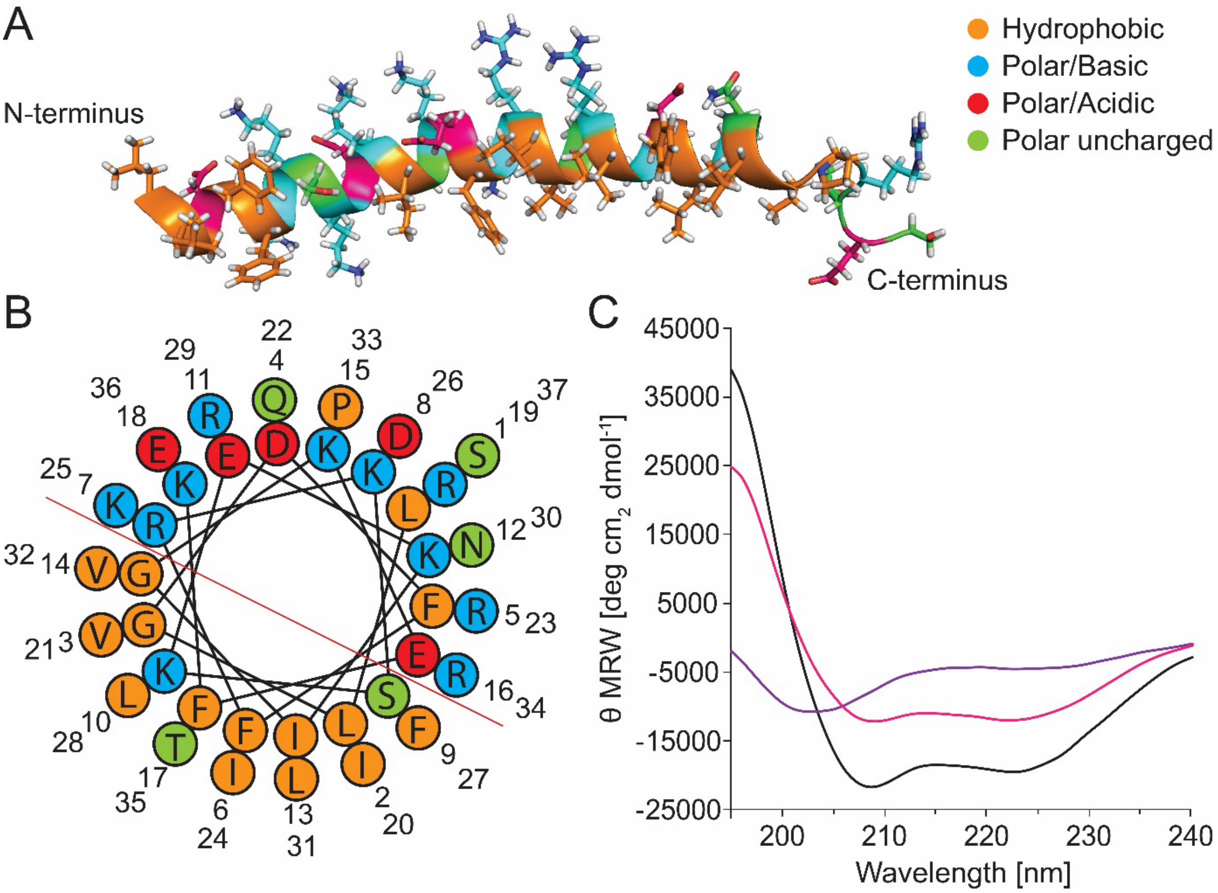

Figure S1: Structure of LL-37. (A) Solution structure of LL-37 in the presence of SDS micelles (pdb ID 2K6O). Hydrophobic (orange), basic (blue), acidic (red) and uncharged polar (green) amino acids are indicated. (B) Helical wheel projection of LL-37 with Heliquest[2]. The hydrophobic and the hydrophilic interfaces are indicated (red line). (C) CD spectra of LL-37 in 20 mM AmAc in the presence (black) and in the absence (purple) of 0.5 % (w/v) C8E4 as well as in 1 x PBS (pink).

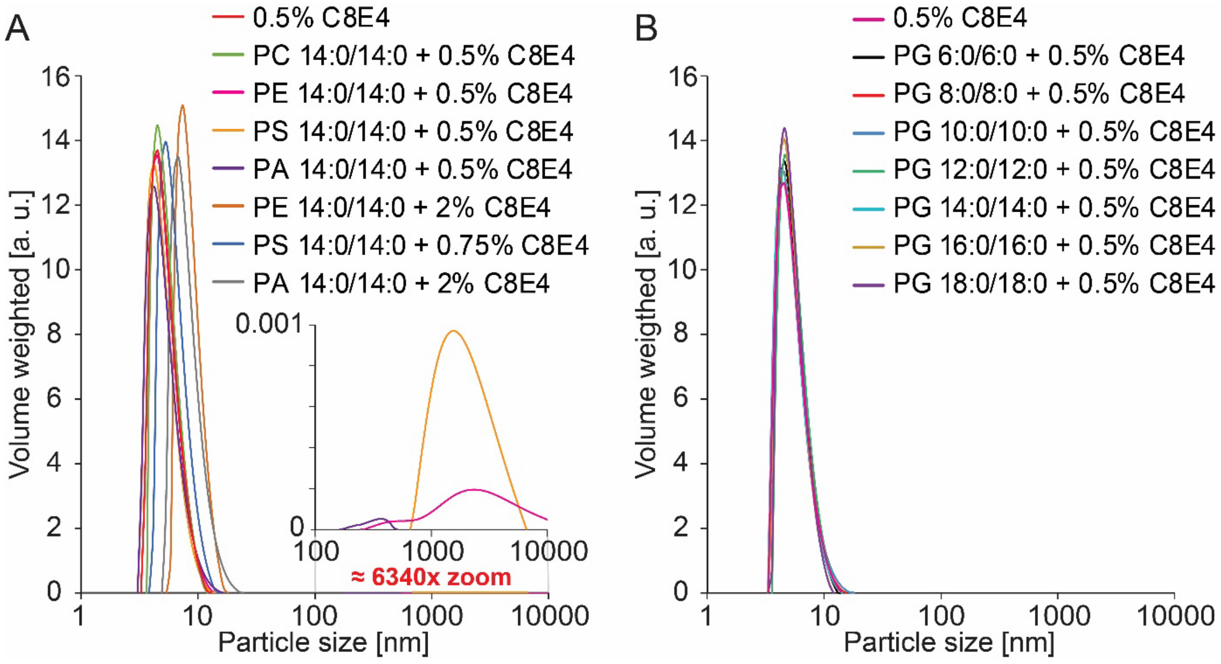

Figure S2: DLS analysis of detergent-lipid micelles. (A) Volume weighted particle size distribution of detergent-lipid micelles containing lipids with different headgroups. A population of larger particles was observed for PS 14:0/14:0, PA 14:0/14:0 and PE 14:0/14:0 (inset). For PS 14:0/14:0, PA 14:0/14:0 and PE 14:0/14:0 particle size distributions at higher C8E4 concentrations are shown (0.75 % (w/v) for PS and 2.0 % (w/v) for PA and PE). (B) Volume weighted particle size distribution of detergent-lipid micelles containing PG-lipids varying in fatty acyl chain lengths.

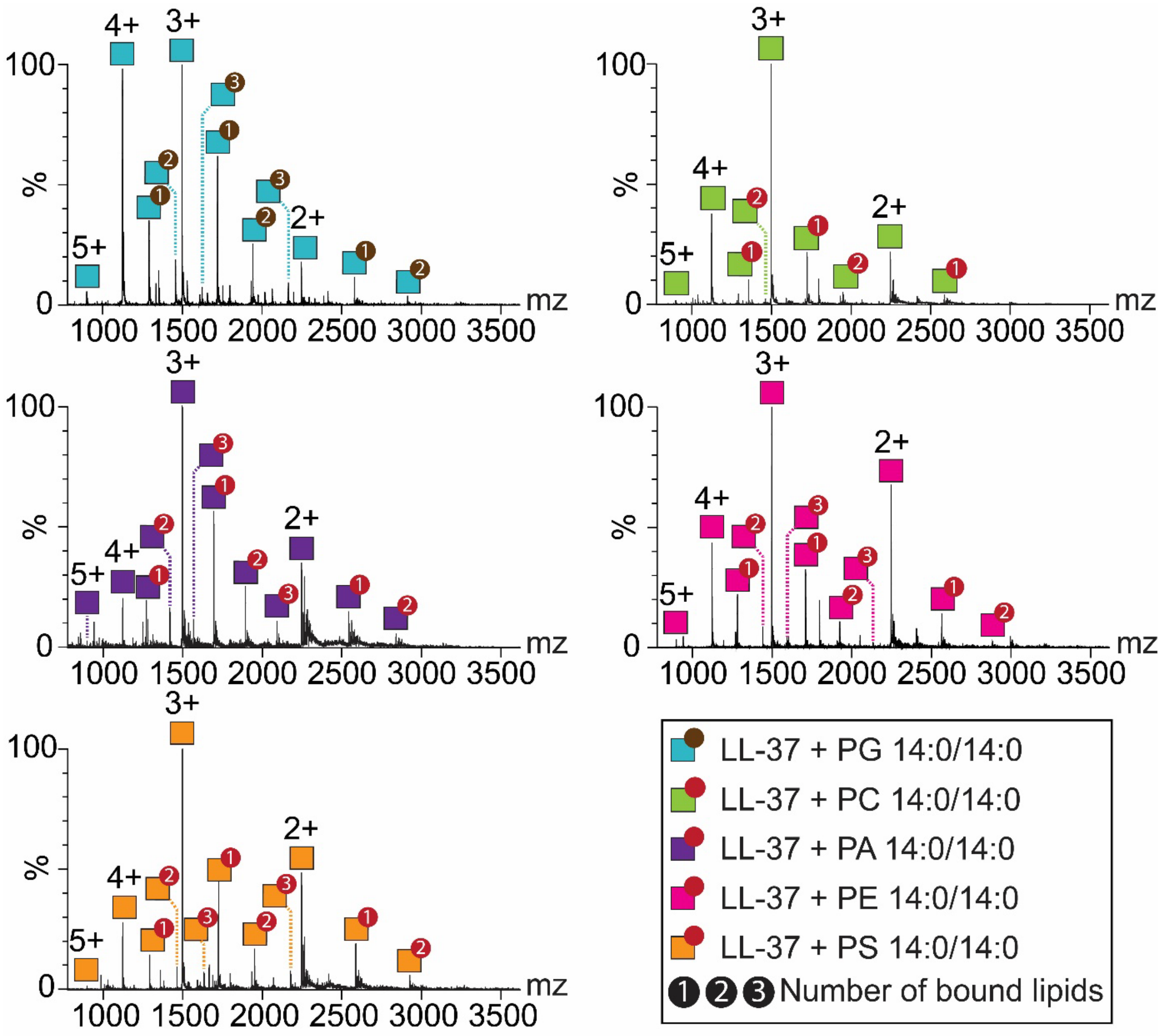

Figure S3: Exploring electrostatic interactions with lipid headgroups. Native MS of 20 µM LL-37 in the presence of 0.5 % (w/v) C8E4 and 25 µM PG 14:0/14:0 (blue), PC 14:0/14:0 (green), PA 14:0/14:0 (purple), PE 14:0/14:0 (pink) or PS 14:0/14:0 (orange). Charge states and lipid adducts are assigned. Masses of LL-37-lipid complexes are given in **Table S1**.

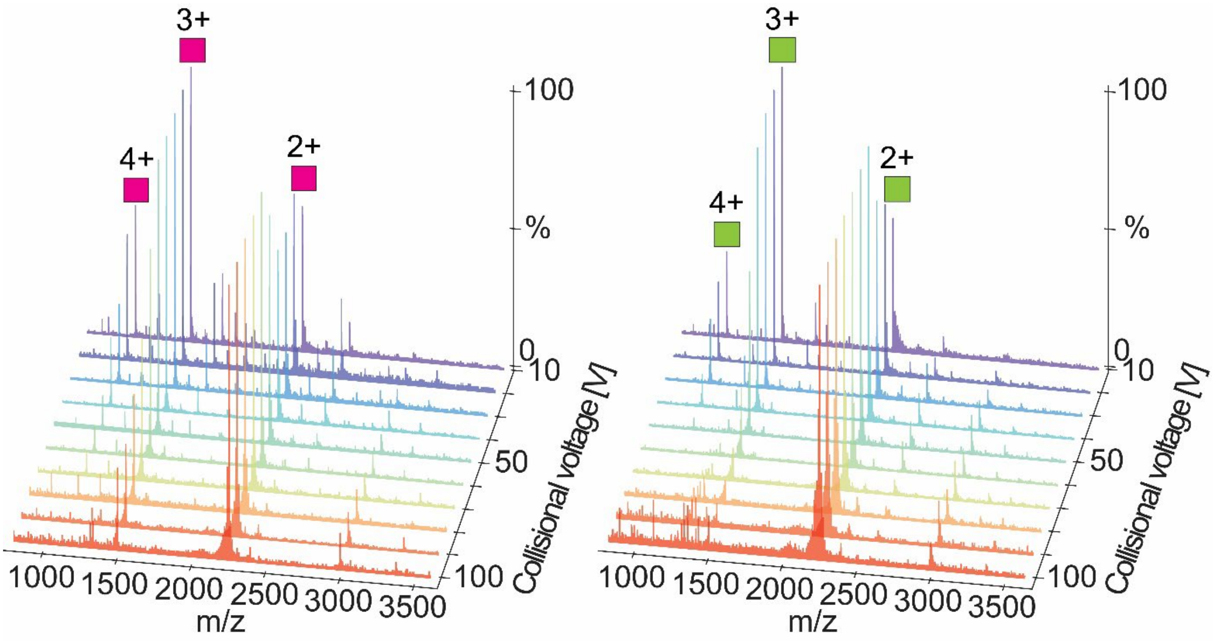

Figure S4: Dissociation of LL-37-PE and LL-37-PC complexes. Native MS of LL-37 in the presence of C8E4 with PE 14:0/14:0 (left) or PC 14:0/14:0 (right) at different collisional voltages. Charge states and peaks corresponding to the LL-37 monomers are assigned. The intensity of LL-37-lipid complexes decreases with increasing collisional voltage. At higher collisional voltages (i.e., above 70 V) an increase in background signal was observed.

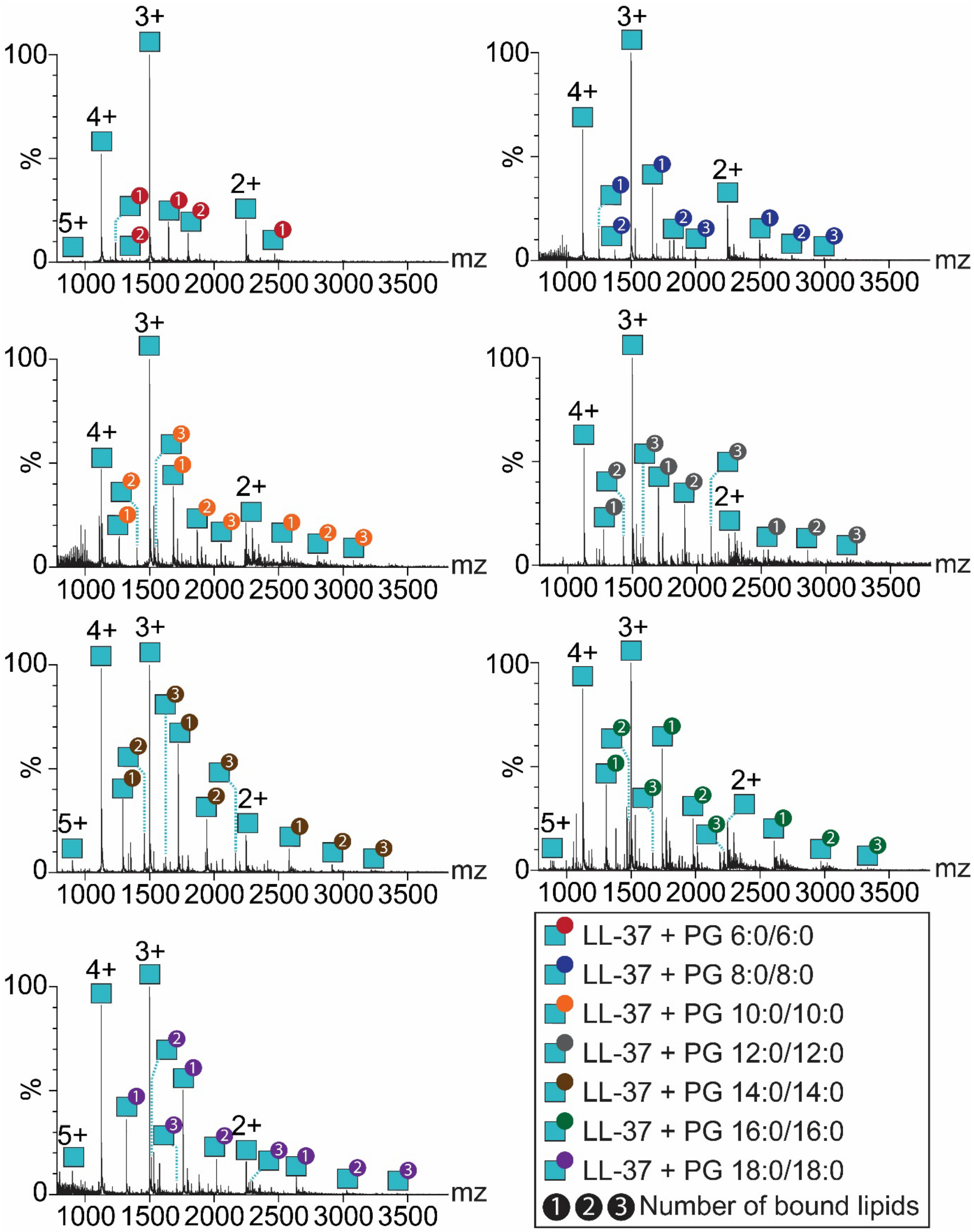

Figure S5: Exploring hydrophobic interactions of LL-37 with fatty acyl chains. 20 μM LL-37 were analyzed in the presence of 0.5 % (w/v) C8E4 and 25 μM PG 6:0/6:0 (red), PG 8:0/8:0 (blue), PG 10:0/10:0 (orange), PG 12:0/12:0 (grey), PG 14:0/14:0 (brown), PG 16:0/16:0 (green) or PG 18:0/18:0 (purple). Charge states and lipid adducts are assigned. Masses of LL-37-lipid complexes are given in **Table S1**.

3. Supporting Tables

Table S1: Masses of LL-37 and LL-37-lipid complexes determined by native MS. The figure number, the peptide and lipid, the theoretical mass and the experimental mass of assigned peaks are given. Theoretical masses were calculated from the molecular weight of associated lipids and the amino acid sequence of LL-37. Experimental masses and the corresponding error were determined using MassLynx v4.1.

Figure	LL-37/ LL-37-lipid complex		Theoretical mass (Da)	Experimental mass (Da)
2	LL-37	10 V	4493.32	4491.64 ± 0.01
	LL-37 + 1 × PS 14:0/14:0	10 V	5171.124	5171.54 ±0.00
	LL-37 + 2 × PS 14:0/14:0	10 V	5849.968	5850.31 ± 0.13
	LL-37 + 3 × PS 14:0/14:0	10 V	6528.812	6531.75 ± 0.00
	LL-37	50 V	4493.32	4491.61 ± 0.03
	LL-37 + 1 × PS 14:0/14:0	50 V	5171.124	5171.96 ± 0.01
	LL-37 + 2 × PS 14:0/14:0	50 V	5849.968	5851.85 ±0.00
	LL-37 + 3 × PS 14:0/14:0	50 V	6528.812	6531.28 ± 0.00
	LL-37	100 V	4493.32	4491.65 ± 0.00
	LL-37 + 1 × PS 14:0/14:0	100 V	5171.124	5171.96 ± 0.03
S3	LL-37		4493.32	4491.69 ± 0.02<br>4491.40 ± 0.16<br>4491.45 ± 0.19<br>4491.68 ± 0.15<br>4491.64 ± 0.01
	LL-37 + 1 × PG 14:0/14:0		5158.125	5159.02 ± 0.03
	LL-37 + 2 × PG 14:0/14:0		5823.97	5825.92 ±0.00
	LL-37 + 3 × PG 14:0/14:0		6489.815	6493.14 ± 0.02
	LL-37 + 1 × PC 14:0/14:0		5170.23	5169.73 ± 0.00
	LL-37 + 2 × PC 14:0/14:0		5848.18	5848.17 ± 0.01
	LL-37 + 1 × PA 14:0/14:0		5084.047	5083.98 ± 0.06
	LL-37 + 2 × PA 14:0/14:0		5675.814	5676.44 ± 0.04
	LL-37 + 3 × PA 14:0/14:0		6267.581	6269.81 ± 0.00
	LL-37 + 1 × PE 14:0/14:0		5128.133	5127.94 ± 0.00
	LL-37 + 2 × PE 14:0/14:0		5763.986	5763.59 ± 0.00
	LL-37 + 3 × PE 14:0/14:0		6399.839	6399.72 ± 0.00
	LL-37 + 1 × PS 14:0/14:0		5171.124	5171.54 ± 0.00
	LL-37 + 2 × PS 14:0/14:0		5849.968	5850.31 ± 0.13
	LL-37 + 3 × PS 14:0/14:0		6528.812	6531.75 ± 0.00
4	LL-37	10 V	4493.32	4491.69 ± 0.02
	LL-37 + 1 × PG 14:0/14:0	10 V	5158.125	5159.02 ± 0.03
	LL-37 + 2 × PG 14:0/14:0	10 V	5823.97	5825.92 ± 0.00
	LL-37 + 3 × PG 14:0/14:0	10 V	6489.815	6493.14 ± 0.02
	LL-37	50 V	4493.32	4491.71 ± 0.07
	LL-37 + 1 × PG 14:0/14:0	50 V	5158.125	5159.06 ± 0.01
	LL-37 + 2 × PG 14:0/14:0	50 V	5823.97	5826.23 ± 0.3

	LL-37 + 3 × PG 14:0/14:0	50 V	6489.815	6493.22 ± 0.00
	LL-37	100 V	4493.32	4491.80 ± 0.03
	LL-37 + 1 × PG 14:0/14:0	100 V	5158.125	5158.00 ± 0.73
S5	LL-37		4493.32	4491.95 ± 0.03<br>4491.49 ± 0.03<br>4491.67 ± 0.00<br>4491.68 ± 0.05<br>4491.69 ± 0.02<br>4491.81 ± 0.19<br>4492.07 ± 0.08
	LL-37 + 1 × PG 6:0/6:0		4933.7	4933.53 ± 0.00
	LL-37 + 2 × PG 6:0/6:0		5375.12	5374.60 ± 0.00
	LL-37 + 1 × PG 8:0/8:0		4989.806	4989.82 ± 0.00
	LL-37 + 2 × PG 8:0/8:0		5487.332	5488.07 ± 0.00
	LL-37 + 3 × PG 8:0/8:0		5984.858	5989.32 ± 0.00
	LL-37 + 1 × PG 10:0/10:0		5045.913	5046.05 ± 0.02
	LL-37 + 2 × PG 10:0/10:0		5599.546	5602.00 ± 0.06
	LL-37 + 3 × PG 10:0/10:0		6153.179	6154.62 ± 0.02
	LL-37 + 1 × PG 12:0/12:0		5102.019	5102.93 ± 0.09
	LL-37 + 2 × PG 12:0/12:0		5711.758	5713.48 ± 0.07
	LL-37 + 3 × PG 12:0/12:0		6321.497	6323.95 ± 0.08
	LL-37 + 1 × PG 14:0/14:0		5158.125	5159.02 ± 0.03
	LL-37 + 2 × PG 14:0/14:0		5823.97	5825.92 ± 0.00
	LL-37 + 3 × PG 14:0/14:0		6489.815	6493.14 ± 0.02
	LL-37 + 1 × PG 16:0/16:0		5214.232	5215.18 ± 0.04
	LL-37 + 2 × PG 16:0/16:0		5936.184	5938.11 ± 0.00
	LL-37 + 3 × PG 16:0/16:0		6658.136	6660.43 ± 0.00
	LL-37 + 1 × PG 18:0/18:0		5270.338	5271.14 ± 0.10
	LL-37 + 2 × PG 18:0/18:0		6048.396	6050.57 ± 0.05
	LL-37 + 3 × PG 18:0/18:0		6826.454	6828.58 ± 0.04

4. Supporting References

(1) Kelly, S. M.; Jess, T. J.; Price, N. C. How to study proteins by circular dichroism. *Biochim Biophys Acta* **2005**, *1751*, 119–139.
(2) Gautier, R.; Douguet, D.; Antonny, B.; Drin, G. HELIQUEST: a web server to screen sequences with specific alpha-helical properties. *Bioinformatics* **2008**, *24*, 2101–2102.
